# Relationship between the burden of major periodontal bacteria and serum lipid profile in a cross-sectional Japanese study

**DOI:** 10.1186/s12903-018-0536-0

**Published:** 2018-05-04

**Authors:** Youn-Hee Choi, Takayuki Kosaka, Miki Ojima, Shinichi Sekine, Yoshihiro Kokubo, Makoto Watanabe, Yoshihiro Miyamoto, Takahiro Ono, Atsuo Amano

**Affiliations:** 1 Department of Preventive Dentistry, School of Dentistry, Kyungpook National University, Daegu, Republic of Korea; 2 Department of Prosthodontics, Gerontology and Oral Rehabilitation, Osaka University Graduate School of Dentistry, Suita-Osaka, Japan; 3 Department of Preventive Dentistry, Osaka University Graduate School of Dentistry, 1-8 Yamadaoka, Suita-Osaka, 565-0871 Japan; 4 Department of Preventive Cardiology, National Cerebral and Cardiovascular Center, Suita-Osaka, Japan; 5 Division of Comprehensive Prosthodontics, Niigata University Graduate School of Medical and Dental Sciences, Niigata, Japan

**Keywords:** Microbiology, Obesity, Periodontal-systemic disease interaction

## Abstract

**Background:**

The association of periodontal bacteria with lipid profile alteration remains largely unknown, although it has been suggested that chronic periodontitis increases the atherosclerotic risk. This cross-sectional study investigated the relationship between the prevalence and total burden of periodontal bacteria and serum lipid profile.

**Methods:**

Saliva from enrolled participants was collected to detect 4 major periodontal bacteria (*Porphyromonas gingivalis, Treponema denticola, Tannerella forsythia,* and *Prevotella intermedia*) using Polymerase Chain Reaction method. High-density lipoprotein (HDL) cholesterol, triglycerides (TG), and low-density lipoprotein cholesterol were assessed using blood samples. We compared the averages of each lipid in association with the prevalence of each bacterial species, their burden (low, moderate, and high), and the combination of bacterial burden and periodontal status, defined as periodontitis, using the Community Periodontal Index, after adjustment for other potential confounding factors, by employing general linear models with least square means.

**Results:**

A total of 385 Japanese individuals (176 men, 209 women; mean age 69.2 years) were enrolled. The number of bacterial species and their co-existence with periodontitis were significantly related to a decrease in HDL (*p* for trend < 0.01) and increase in TG (*p* for trend = 0.04). The adjusted mean HDL levels (mg/dL) in individuals with low, moderate, and high levels of bacterial species were 66.1, 63.0, and 58.9, respectively, and those in the 6 groups defined by combination of the two factors were 67.9, 64.6, 64.3, 65.4, 61.5, and 54.7, respectively.

**Conclusion:**

Periodontal bacterial burden is suggested to be independently involved in lowering serum HDL level. Our findings suggest that bacterial tests in a clinical setting could be a useful approach for predicting the risk of HDL metabolism dysregulation.

## Background

Recent studies have revealed that chronic marginal periodontitis is associated with atherosclerosis (as an intermediate endpoint) and cardiovascular diseases [1–3]. Periodontitis and its severity have been operationally defined by two types of clinical measurements, i.e., pocket depth and/or clinical attachment loss, in population studies [4, 5]. The clinical definition of periodontitis is dependent on the patient’s condition or the characteristics of the target study population, without any established gold standard. Thus, more valid, more reliable, and various measures for the chronic periodontitis associated with cardiovascular diseases seems to be required. Previous studies have suggested that the serum antibody levels against specific periodontal bacteria, such as *Porphyromonas gingivalis* and *Aggregatibacter actinomycetemcomitans*, or periodontitis-related microorganism groups [6] are useful to predict the risk of atherosclerotic diseases [7–9]. Furthermore, periodontal bacteria harbored in gingival pockets, in regard to the burden of total bacterial species possessed by an individual, has been suggested to be a risk factor for atherosclerosis [10] and myocardial infarction [1].

As for major periodontal pathogenic bacteria, *P. gingivalis*, *Prevotella intermedia*, and *Treponema denticola*, as well as several other species, have been suggested to be possible causative agents in an in vivo study of atherosclerosis [11]. Another recent study [12] showed that specific bacterial groups, including the Orange-Red complex of *P. intermedia, P. gingivalis, T. denticola,* and *Tannerella forsythia*, were associated with elevated plasma glucose levels in adults. However, it did not show that those bacterial groups were related to other metabolic syndrome components. In contrast, another recent study [13] suggested that periodontitis, as determined by pocket depth, clinical insertion level, and bleeding on probing, was associated with distorted serum lipid levels, and also noted that the serum levels of *P. gingivalis* and *A. actinomycetemcomitans* antibodies may be a risk factor for decrease high-density lipoprotein (HDL) cholesterol levels. In light of metabolic pathways, *P. gingivalis* and its vesicles, for example, promote multiple cytokines as chronic infection and inflammation status resulting in elevation of triglyceride rich lipoprotein affecting on other lipoproteins. In turn, increase of triglyceride raises low-density lipoprotein (LDL) binding to macrophages and induce macrophages to modify native LDL, which plays an important role in foam cell formation [14, 15]. Notwithstanding, the association of periodontal pathogenic bacteria with serum lipid profile alteration as an atherosclerotic risk factor has not been well investigated, even though periodontitis is thought to be tightly related to the development of atherosclerosis. In addition, the effects of periodontal destruction, as a clinical parameter of the relationship between exposure to periodontal bacteria and lipid profiles in humans, are not well known. Therefore, in the present study, we hypothesized that exposure to periodontal bacteria could alter serum lipid metabolic pathways. We aimed to investigate the relationship between the prevalence of periodontal bacterial species with the total periodontal bacterial burden and the serum lipid profile, and to assess the combined effects of bacterial factors and the presence of periodontitis on the level of serum lipids.

## Methods

### Study participants

**Fig. 1:**
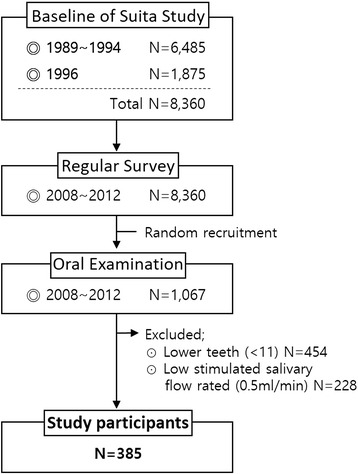
Flow chart of the process of recruitment for the final study participants

We recruited 1067 Japanese individuals aged 30–79 years who underwent a medical check-up and oral examination between June 2008 and March 2010 as part of the Suita Study, which comprised a random sample of 8360 Japanese urban residents. The Suita Study was originally constructed as a cohort between September 1989 and March 1994, and a regular health examination was performed every 2 years between June 2008 and March 2012. In the beginning of the Suita Study, 6485 of the 12,200 registered residents of Suita City underwent general health checkups at the National Center for Cerebral and Cardiovascular diseases [16]. In 1996, a second recruitment commenced, and 1875 additional individuals underwent basic health examinations, as shown in Fig. 1.

Oral health examination especially for periodontal pathogenic bacterial assessment was underwent as a cross-sectional design for the follow-up period of time. Prior to enrollment, the study protocol was approved by the Ethics Committee of the National Cerebral and Cardiovascular Center (M25–032), and only individuals who provided informed consent after receiving a full explanation of the study purpose and methods, both in writing and orally, were included as study participants.

Inclusion criteria for every participant was the presence of 10 teeth and more in mouth. From a total of 1067, 454 persons were excluded in this study because they had under 10 teeth in their mouth. Among the rest of the people (*n* = 613), individuals were excluded if they had low stimulated salivary flow rate (< 0.5 ml min^− 1^) (*n* = 228). Thus, final sample size was 385.

### Assay for bacterial analysis in saliva

The volunteers were asked to chew a piece of paraffin gum for 2 min to stimulate salivary flow, and then spit the saliva into a 50 ml Falcon test tube (Corning Inc., Corning, NY, USA), as described previously [17]. After collection, the volume of each saliva sample was determined gravimetrically, and the stimulated salivary flow rates were expressed as ml min^− 1^. Samples were stored at − 80 °C until required for analysis. Saliva samples were collected prior to periodontal tissue examination in order to avoid possible side effects.

Bacterial genomic DNA was isolated from the specimens using a Wizard^®^ Genomic DNA Purification Kit (Promega Corporation, Madison, WI, USA), according to the manufacturer’s instructions. A 16S RNA-based polymerase chain reaction (PCR) detection method was used to determine the prevalence of four periodontal bacteria (*P. gingivalis, T. denticola, T. forsythia, P. intermedia*), as previously described [18–21]. Table 1 lists the PCR primers used in the study. PCR amplification was performed in a total volume of 25 μL consisting of DNA polymerase (SpeedSTAR^®^ HS DNA Polymerase, Takara Bio Inc., Shiga, Japan), 0.5 μM each primer, 10× PCR buffer, dNTP mixture, and 1 μL of the template DNA solution in sterile distilled water. PCR was performed in a thermal cycler (2720 Thermal Cycler, Applied Biosystems, Foster City, CA, USA) with the following cycling parameters: initial denaturation at 95 °C for 2 min, followed by 30 cycles consisting of 95 °C for 10 s and 62 °C for 30 s. Positive and negative controls were included in each PCR set, and when processing all samples. PCR products were analyzed by 2.0% agarose gel (Agarose S, Nippon Gene, Tokyo, Japan) electrophoresis. The gel was stained with 0.5 μg/mL ethidium bromide (Nippon Gene, Tokyo, Japan) and photographed under UV illumination. A 100-bp DNA ladder (EZ Load™, BioRad, CA, USA) was used as a molecular size standard. The detection limit was determined for the simultaneous PCR using known numbers of bacterial cells diluted in distilled water.

**Table 1:** List of Species-specific primers

Primer set	Sequence (5′ to 3′)	Size (bp)	Detection Limit (No. of cells)	Reference
P. gingivalis	TGT AGA TGA CTG ATG CTG AAA ACC	197	5	(17)
	ACG TCA TCC CCA CCT TCC TC			
T. denticola	AAG GCG GTA GAG CCG CTC A	311	10	(18)
	AGC CGC TGT CGA AAA GCC CA			
T. forsythia	GCG TAT GTA ACC TGC CCG CA	641	25	(15)
	TGC TTC AGT GTC AGT TAT ACC T			
*P. intermedia*	TTTGTTGGGGAGTAAAGCGGG	575	25	(15)
	TCAACATCTCTGTATCCTGCGT			

### Periodontal tissue examination and oral health behavior

Periodontal tissue examinations were performed by 5 dentists (Y. Y., K. T., M. K., T. K., and M. K.) who were appropriately trained and calibrated according to standardized procedures as recommended in the manual published by the World Health Organization [22]. For each individual, a total of 10 teeth were examined, the maxillary and mandibular left and right first and second molars, maxillary right central incisor, and mandibular left central incisor. When an examination could not be performed because one or both the central incisors was missing, the same tooth on the opposite side was examined. No evaluation was performed when all relevant teeth were missing. Periodontal status was examined at 6 sites of each tooth using a Community Periodontal Index (CPI) probe (YDM, Tokyo, Japan) according to the following criteria, with the highest-value code recorded. The CPI codes were as follows: Code 0, no sign of gingival inflammation; Code 1, bleeding evident after probing; Code 2, dental calculus deposits detected (including those detected by probing up to 4 mm beneath the gingival margin); Code 3, periodontal pocket depth ranging from ≥ 4 mm to < 6 mm; Code 4, periodontal pocket depth ≥ 6 mm. Cohen’s κ value for consistency between the periodontal tissue examinations performed by the 5 dentists was 0.78. Clinical periodontitis was defined as a CPI code greater than 2. Information about frequency of tooth brushing (once, twice, more than twice per day) and dental flossing (none, once or more per week, once or more per day) was also used.

## Laboratory testing of serum for lipid profile and other general health information

Routine blood tests were used to measure levels of HDL, triglycerides (TG), and LDL cholesterol in serum, with total cholesterol (TC) levels calculated using the following formula: HDL + LDL + TG/5. These 4 variables (HDL, TG, LDL, and TC) were used as outcomes. Fasting glucose level was also assessed to define diabetes (fasting glucose ≥126 mg/L, or use of diabetic medication). Body mass index (BMI) was calculated by measurement of height and body weight [23]. Well-trained physicians measured systolic (SBP) and diastolic (DBP) blood pressure. Hypertension was defined as SBP ≥ 140 mmHg and/or DBP ≥ 90 mmHg or use of antihypertensive treatment [24]. In addition, lifestyle details, such as smoking, drinking, and regular exercise, were obtained using a standardized questionnaire by trained physicians or nurses in face-to-face interviews, as previously described [24]. Smoking and drinking were further classified into current, former, and never. The number of times of exercising per week was categorized as none, once or twice, and more than twice.

## Statistical methods

Exposure was considered as the total number of periodontal bacteria and the presence of bacteria themselves, and the outcome was considered as each type of serum lipid. Primary exposure and outcome were the number of periodontal pathogens found in saliva, defined as the bacterial burden and the level of HDL, respectively. To understand the demographic, behavioral, and oral health-related factors in study participants according to bacterial type, we performed bivariate analyses of covariates, including age, sex, smoking, drinking, exercise, tooth brushing, dental flossing, periodontitis, number of existing teeth, and the DMFT (decayed, missing, and filled) index according to the 4 bacteria and burden level. For this, chi-square tests or *t*-tests were performed with SAS version 9.4 (SAS Institute Inc. Cary, NC, USA). The threshold for statistical significance was set at 0.05.

In terms of the operational definition of bacterial burden, the total bacterial burden of periodontal pathogenicity in this study was defined in cases in which the 4 types of periodontal bacteria were found in the saliva. A person was classified as having a high bacterial burden when all 4 bacteria were found in his/her saliva, which indicates a relatively higher exposure to periodontal pathogens. Therefore, the presence of a bacterial species scored 1 point for any of the 4 periodontal bacteria; exposure was considered as low (none of the 4 bacterial species present), moderate (presence of 1–3 bacterial species), or high (all bacterial species present) for statistical analyses.

In second step, to evaluate the crude association between the presence of periodontal bacteria and bacterial burden with periodontitis, and 4 types of serum lipid profiles, bivariate analyses were tested using *t*-test or one-way Analysis of Variance (ANOVA) with post hoc analysis. Bacterial pathogenicity was measured from not gingival crevicular fluid but stimulated saliva that is easier to quickly collect from many people. In addition, the saliva could include all bacteria originated from the whole periodontal sites of total teeth in mouth.

In order to measure the statistically independent strengths of the association of bacterial burden on lipids, general linear models (GLM) were constructed after adjustment for potential confounding variables, including age, sex, periodontitis, BMI, diabetes, hypertension, smoking, drinking, exercise, tooth brushing, and dental flossing. To observe the change in effect size, confounders were added step by step into the models and the least square mean (LSM) values for lipid levels were also employed. Their *p* for trend values were tested as well. Lastly, LSMs were calculated again for the 6 groups categorized by bacterial strains and periodontitis after adjustment for age, sex, BMI, diabetes, hypertension, smoking, drinking, exercise, tooth brushing, and dental flossing for the combined effect on lipid level. The *p* for trend values were also determined.

Regarding of GLM analysis, the distribution of lipids variables was initially checked whether or not they were normal. The lipids were not normally distributed as a matter of fact so that they were transformed by square root, inverse square root, and natural log. The transformed results were compared to those of GLM. For preference of robust model, GLM analysis was finally selected. Normality test for outcomes, Shapiro-Wilk, Kolmogorov-Smirnov, Cramer-von Mises and Anderson-Darling tests were used for each 4 lipid type. And then the outcomes were transformed by square root, inverse square root, and natural log. The findings of GLM analysis in transformed data were compared to those in unconverted data (not shown).

## Results

As shown in Table 2, we examined 385 Japanese study participants (176 men and 209 women, average age 69.2 years). Those with greater numbers of different species of periodontal bacteria were more likely to be a current or ex-smoker (*p* = 0.05) and had periodontitis (*p* < 0.01). Periodontitis group showed a tendency to possess all of the periodontal bacteria, while *P. intermedia* was more likely to be harbored by ex-smokers and a higher percentage of older people possessed *P. gingivalis*.

**Table 2:** Characteristics of demographic, behavioral, and oral health-related factors according to bacterial type

		Prevalence of periodontal bacteria								Periodontal pathogenic burden		
Variable name	Total	*P. gingivalis*		*T. denticola*		*T. forsythia*		*P. intermedia*				
		Yes	No	Yes	No	Yes	No	Yes	No	Low	Moderate	High
	*No. (%)*											
Total	385 (100.0)	224(58.2)	161(41.8)	230(59.7)	155(40.3)	285(74.0)	100(26.0)	203(52.7)	182(47.3)	46(11.9)	230(59.7)	109(28.3)
Age (y)												
50−59	**51 (13.2)**	**20(8.9)**	**31 (19.3)**	30 (13.0)	21 (13.5)	37 (13.0)	14 (14.0)	27 (13.3)	24 (13.2)	5 (10.9)	39 (17.0)	7 (6.4)
60 −69	**108 (28.1)**	**64 (28.6)**	**44 (27.3)**	68 (29.6)	40 (25.8)	86 (30.2)	22 (22.0)	57 (28.1)	51 (28.0)	10 (21.7)	64 (27.8)	34 (31.2)
70−82	**226 (58.7)**	**140 (62.5)**	**86 (53.4)**	132 (57.4)	94 (60.6)	162 (56.8)	64 (64.0)	119 (58.6)	107 (58.8)	31 (67.4)	127 (55.2)	68 (62.4)
*p-value*		**0.01**		0.72		0.29		1.00		0.07		
Gender												
Male	176 (45.7)	108 (48.2)	68 (42.2)	106 (46.1)	70 (45.2)	136 (47.7)	40 (40.0)	98 (48.3)	78 (42.9)	16 (34.8)	107 (46.5)	53 (48.6)
Female	209 (54.3)	116 (51.8)	93 (57.8)	124 (53.9)	75 (54.8)	149 (52.3)	60 (60.0)	105 (51.7)	104 (57.1)	30 (65.2)	123 (53.5)	56 (51.4)
*p-value*		0.25		0.86		0.18		0.30		0.27		
Smoking												
Current	31 (8.1)	18 (9.2)	13 (9.1)	16 (7.7)	15 (11.5)	22 (8.6)	9 (10.8)	**15 (8.2)**	**16 (10.3)**	**2 (5.1)**	**23 (11.5)**	**6 (6.0)**
Former	100 (26.0)	64 (32.7)	36 (25.2)	67 (32.1)	33 (25.4)	82 (32.0)	18 (21.7)	**65 (35.5)**	**35 (22.4)**	**7 (17.9)**	**55 (27.5)**	**38 (38.0)**
Never	208 (54.0)	114 (58.2)	94 (65.7)	126 (60.3)	82 (63.1)	152 (59.4)	56 (67.5)	**103 (56.3)**	**105 (67.3)**	**30 (76.9)**	**122 (61.0)**	**56 (56.0)**
*p-value*		0.31		0.27		0.19		**0.03**		**0.05**		
Drinking												
Current	144 (37.4)	90 (45.9)	54 (37.8)	88 (42.1)	56 (43.1)	115 (44.9)	29 (34.9)	77 (42.1)	67 (42.9)	13 (33.3)	87 (43.5)	44 (44.0)
Former	19 (4.9)	11 (5.6)	8 (5.6)	13 (6.2)	6 (4.6)	14 (5.5)	5 (6.0)	9 (4.9)	10 (6.4)	2 (5.1)	12 (6.0)	5 (5.0)
Never	176 (54.0)	95 (48.5)	81 (56.6)	108 (51.7)	68 (52.3)	127 (49.6)	49 (59.0)	97 (53.0)	79 (50.6)	24 (61.5)	101 (50.5)	51 (51.0)
*p-value*		0.31		0.82		0.28		0.80		0.77		
Exercise (times/week)												
None	110 (28.6)	57 (29.2)	53 (37.1)	68 (32.7)	42 (32.3)	84 (32.9)	26 (31.3)	65 (35.7)	45 (28.8)	16 (41.0)	59 (29.5)	35 (35.4)
≤ Twice	228 (59.2)	138 (70.8)	90 (62.9)	140 (67.3)	88 (67.7)	171 (67.1)	57 (68.7)	117 (64.3)	111 (71.2)	23 (59.0)	141 (70.5)	64 (64.6)
*p-value*		0.13		0.94		0.79		0.18		0.29		
Brushing frequency (times/day)												
Once	75 (19.5)	40 (21.1)	35 (26.7)	46 (22.5)	29 (24.8)	58 (23.4)	17 (23.3)	43 (23.9)	32 (22.7)	9 (28.1)	41 (21.6)	25 (25.3)
Twice	163 (42.3)	96 (50.5)	67 (51.1)	102 (50.0)	61 (52.1)	127 (51.2)	36 (49.3)	93 (51.7)	70 (49.6)	15 (46.9)	101 (53.2)	47 (47.5)
≥ 3 times	83 (21.6)	54 (28.4)	29 (22.1)	56 (27.5)	27 (23.1)	63 (25.4)	20 (27.4)	44 (24.4)	39 (27.7)	8 (25.0)	48 (25.3)	27 (27.3)
*p-value*		0.32		0.68		0.94		0.81		0.58		
Flossing frequency												
None	142 (36.9)	80 (41.7)	62 (45.9)	93 (45.4)	49 (40.2)	109 (43.6)	33 (42.9)	81 (45.0)	61 (41.5)	16 (47.1)	78 (40.0)	48 (49.0)
≥ Once/week	55 (14.3)	35 (18.2)	20 (14.8)	29 (14.1)	26 (21.3)	43 (17.2)	12 (15.6)	28 (15.6)	27 (18.4)	5 (14.7)	37 (19.0)	13 (13.3)
≥ Once/day	130 (33.8)	77 (40.1)	53 (39.3)	83 (40.5)	47 (38.5)	98 (39.2)	32 (41.6)	71 (39.4)	59 (40.1)	13 (38.2)	80 (41.0)	37 (37.8)
*p-value*		0.64		0.24		0.91		0.73		0.85		
Periodontitis by CPI*												
Periodontitis(−)	195 (54.2)	**105 (48.8)**	**90 (62.1)**	**100 (44.4)**	**95 (70.4)**	**133 (48.5)**	**62 (72.1)**	**89 (45.2)**	**106 (65.0)**	**27 (75.0)**	**127 (58.5)**	**41 (38.3)**
Periodontitis(+)	165 (45.8)	**110 (51.2)**	**55 (37.9)**	**125 (55.6)**	**40 (29.6)**	**141 (51.5)**	**24 (27.9)**	**108 (54.8)**	**57 (35.0)**	**9 (25.0)**	**90 (41.5)**	**66 (61.7)**
*p-value*		**0.01**		**<.01**		**<.01**		**<.01**		**<.01**		
	*mean ± sd*											
Age (y)	69.2 ± 7.8	69.8 ± 7.2	68.4 ± 8.4	69.1 ± 7.9	69.3 ± 7.7	68.9 ± 7.8	70.1 ± 7.6	69.3 ± 7.8	69.1 ± 7.83	**71.0 ± 8.0** ^**a**^	**68.3 ± 7.9** ^**b**^	**70.3 ± 7.2b** ^**a**^
*p-value*		0.08		0.78		0.18		0.72		**0.05**		
CPI	1.7 ± 1.6	1.8 ± 1.6	1.5 ± 1.5	**2.1 ± 1.5**	**1.1 ± 1.5**	**1.9 ± 1.5**	**1.1 ± 1.5**	**2.0 ± 1.5**	**1.3 ± 1.5**	**1.0 ± 1.5** ^**a**^	**1.6 ± 1.** ^**5a**^	**2.2 ± 1.5** ^**b**^
*p-value*		0.08		**<.01**		**<.01**		**<.01**		**<.01**		
No. of present teeth	20.9 ± 7.8	20.8 ± 7.1	21.1 ± 8.7	**21.7 ± 6.4**	**19.8 ± 9.4**	21.2 ± 7.1	20.1 ± 9.5	21.6 ± 6.8	20.1 ± 8.8	18.8 ± 11.0	21.2 ± 7.6	21.2 ± 6.5
*p-value*		0.70		**0.03**		0.32		0.06		0.22		
DFMT index	18.0 ± 7.1	18.1 ± 7.0	17.8 ± 7.2	18.2 ± 6.8	17.6 ± 7.4	18.1 ± 7.0	17.5 ± 7.1	17.7 ± 6.8	18.3 ± 7.4	18.1 ± 8.1	18.0 ± 6.8	17.9 ± 7.1
*p-value*		0.75		0.38		0.43		0.38		0.43		

Bold indicates statistical significance shown by chi-square test or t- test (*P-value* < 0.05). Total results for each variable may be different due to missing values

Means with the same letter (a and b) are not significantly different by Tukey test. *Community Periodontal Index

Table 3 shows the crude associations between the periodontal bacteria and lipid levels. There were significant relationships between the average HDL level and the presence of each bacterium, except for *P. gingivalis*, the presence of 4 bacteria, and the co-existence of bacteria and clinical periodontitis. Furthermore, the concentration of TG was shown to be significantly elevated along with the increase in the bacterial burden.

**Table 3:** Serum lipid profile due to presence of periodontal bacteria, total pathogenic burden, and periodontitis

Exposures	No. (%)	High-density lipoprotein	Triglycerides	Low-density lipoprotein	Total cholesterol
		mean ± SD	mean ± SD	mean ± SD	mean ± SD
*P. gingivalis*					
Yes	196 (57.8)	60.7 ± 14.6	108.9 ± 64.9	123.3 ± 28.1	205.8 ± 30.4
No	143 (42.2)	63.0 ± 17.3	101.6 ± 50.7	120.0 ± 29.2	203.3 ± 32.8
*p*-value		0.21	0.26	0.30	0.47
*T. denticola*					
Yes	209 (61.7)	**59.8 ± 15.0**	110.1 ± 65.0	121.9 ± 27.0	203.7 ± 30.2
No	130 (38.3)	**64.8 ± 16.8**	98.9 ± 48.4	121.8 ± 31.1	206.4 ± 33.4
*p*-value		**0.00**	0.07	0.96	0.45
*T. forsythia*					
Yes	**256 (75.5)**	**60.7 ± 15.7**	**108.7 ± 64.3**	121.8 ± 28.5	204.3 ± 30.6
No	**83 (24.5)**	**64.7 ± 15.9**	**97.0 ± 39.5**	122.0 ± 28.9	206.0 ± 34.1
*p*-value		**0.05**	**0.05**	1.00	0.66
*P. intermedia*					
Yes	183 (54.0)	**59.9 ± 15.6**	110.6 ± 67.2	122.4 ± 27.5	204.4 ± 29.9
No	156 (46.0)	**63.9 ± 15.8**	100.3 ± 48.2	121.2 ± 29.8	205.1 ± 33.3
*p*-value		**0.02**	0.11	0.68	0.84
Periodontal pathogenic burden					
Low	39 (11.5)	**66.2 ± 16.1a**	97.1 ± 44.3	124.1 ± 27.7	209.7 ± 30.2
Moderate	200 (59.0)	**63.3 ± 16.2a**	102.1 ± 51.9	119.7 ± 29.5	203.4 ± 32.8
High	100 (29.5)	**56.7 ± 13.8b**	116.8 ± 75.4	125.3 ± 26.9	204.7 ± 31.4
*p*-value		**<.01**	0.08	0.19	0.51
Groups based on combination of bacterial burden and periodontitis					
Periodontitis(−) and low	27 (7.0)	**69.5 ± 15.4** ^**a**^	**89.6 ± 43.8** ^**a**^	128.8 ± 31.2	216.1 ± 30.5
Periodontitis(−) and moderate	127 (33.0)	**64.9 ± 16.0** ^**a**^	**91.9 ± 41.0** ^**a**^	119.4 ± 28.7	202.7 ± 30.7
Periodontitis(−) and high	41 (10.6)	**64.1 ± 13.1** ^**a**^	**92.5 ± 39.7** ^**a**^	123.8 ± 19.3	206.4 ± 22.3
Periodontitis(+) and low	9 (2.3)	**63.4 ± 13.1** ^**a**^	**107.0 ± 49.1** ^**a**^	112.7 ± 16.4	197.5 ± 28.4
Periodontitis(+) and moderate	90 (23.4)	**61.9 ± 16.6** ^**ab**^	**113.8 ± 63.2** ^**ab**^	118.4 ± 31.0	203.0 ± 35.6
Periodontitis(+) and high	66 (17.1)	**52.8 ± 12.7** ^**b**^	**131.5 ± 87.1** ^**b**^	125.8 ± 30.7	204.8 ± 32.95
*p*-value		**<.01**	**<.01**	0.38	0.52

*P*-values by ANOVA test or t- test. Means with the same letter (a and b) are not significantly different by Tukey test

**Fig. 2:**
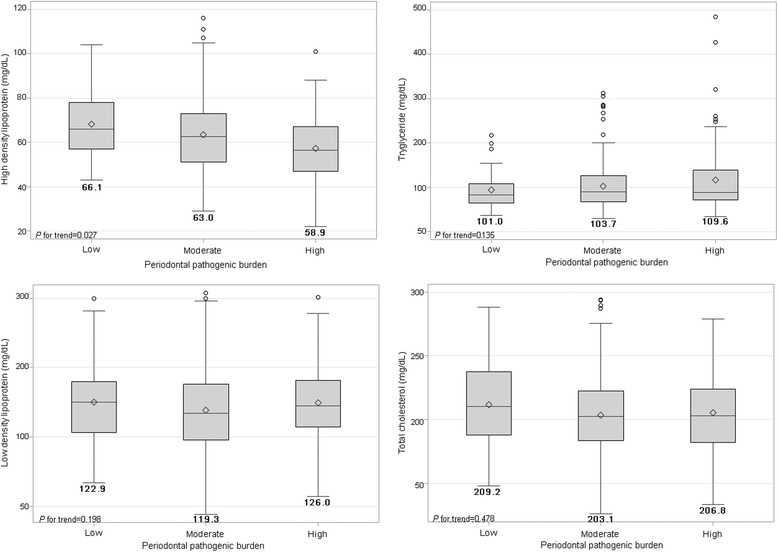
Estimated mean values of serum lipid profiles according to the level of exposure to a periodontal pathogenic burden. Bold numbers under boxes are LSM values of lipids in accordance with the severity of bacterial burden after adjustment for age, sex, periodontitis, BMI, diabetes, hypertension, smoking, drinking, exercise, tooth brushing, and dental flossing frequency

**Fig. 3:**
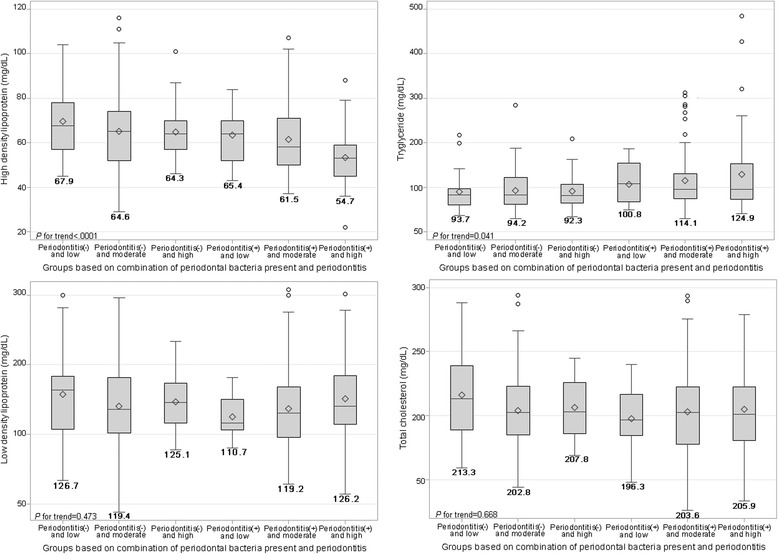
Estimated mean values of high-density lipoprotein (HDL) and triglycerides (TG) due to combined effects of periodontal pathogenic burden and periodontitis. Bold numbers under boxes are LSM values of HDL and TG after adjustment for age, sex, BMI, diabetes, hypertension, smoking, drinking, exercise, tooth brushing, and dental flossing frequency

In multivariable analysis, the presence of all 4 bacterial species was negatively associated with HDL, after adjustment for clinically defined periodontitis and other potential confounding factors, as shown in Table 4 and Fig. 2. Adjusted mean HDL levels according to the bacterial burn group (low, moderate, and high) were significantly reduced (*p* for trend = 0.05) (Table 4). Co-existing periodontal bacteria and clinical periodontitis (periodontitis [−] and periodontitis [+]) was significantly related to decreased HDL (Fig. 2). The adjusted mean HDL levels in patients with a combination of periodontitis (−) and bacterial burden (low, moderate, and high) and periodontitis (+) with the presence of bacterial species (low, moderate, and high) were decreased (*p* for trend < 0.5), while TG levels in those groups showed a trend to increase (*p* for trend < 0.5) as shown in Fig. 3. In reference to transformed lipids analysis, the final results were similar to those shown in Figs. 2 and 3, even though the estimated mean values were slightly different (not shown).

**Table 4:** Means of serum lipids by periodontal pathogenic burden using multivariable GLM models

	Means of serum lipids					
	Model 1		Model 2		Model 3	
	MEAN ± SD	LSM	MEAN ± SD	LSM	MEAN ± SD	LSM
High-density lipoprotein (mg/dL)						
*Periodontal pathogenic burden*						
Low	**66.2 ± 16.1**	65.4	**68.1 ± 14.9**	66.4	**68.1 ± 14.9**	66.1
Moderate	**63.3 ± 16.2**	63.4	**63.6 ± 16.3**	63.1	**63.5 ± 16.3**	63.0
High	**56.7 ± 13.8**	56.9	**56.8 ± 13.9**	58.2	**57.3 ± 13.8**	58.9
	*P* for trend > 0.05		*P* for trend = 0.07		*P* for trend = 0.05	
	Adjusted *R*^2^ = 0.10		Adjusted *R*^2^ = 0.18		Adjusted *R*^2^ = 0.19	
Triglycerides (mg/dL)						
*Periodontal pathogenic burden*						
Low	97.1 ± 44.3	100.6	93.6 ± 44.8	100.0	93.6 ± 44.8	101.0
Moderate	102.1 ± 51.9	101.4	101.5 ± 52.7	102.9	101.8 ± 53.1	103.7
High	116.8 ± 75.4	116.8	117.6 ± 75.8	112.9	115.9 ± 76.1	109.6
	*P* for trend > 0.05		*P* for trend > 0.05		*P* for trend > 0.05	
	Adjusted *R*^2^ = 0.05		Adjusted *R*^2^ = 0.12		Adjusted *R*^2^ = 0.14	
Low-density lipoprotein (mg/dL)						
*Periodontal pathogenic burden*						
Low	124.1 ± 27.7	123.4	125.1 ± 29.1	123.5	125.1 ± 29.1	122.9
Moderate	119.7 ± 29.5	119.6	119.0 ± 29.7	118.9	119.5 ± 29.8	119.3
High	125.3 ± 26.9	125.8	125.1 ± 27.1	125.7	124.9 ± 27.6	126.0
	*P* for trend > 0.05		*P* for trend > 0.05		*P* for trend > 0.05	
	Adjusted *R*^2^ = 0.14		Adjusted *R*^2^ = 0.03		Adjusted *R*^2^ = 0.03	
Total Cholesterol (mg/dL)						
*Periodontal pathogenic burden*						
Low	209.7 ± 30.2	208.9	211.9 ± 30.6	209.9	211.9 ± 30.6	209.2
Moderate	203.4 ± 32.8	203.2	202.8 ± 32.9	202.6	203.3 ± 32.8	203.1
High	205.4 ± 29.2	206.1	205.5 ± 29.5	206.5	205.4 ± 30.1	206.8
	*P* for trend > 0.05		*P* for trend > 0.05		*P* for trend > 0.05	
	Adjusted *R*^2^ = 0.04		Adjusted *R*^2^ = 0.03		Adjusted *R*^2^ = 0.02	

Model 1: Adjusted for age and gender, Model 2: Additionally adjusted for periodontitis, BMI, diabetes, and hypertension, Model 3: Additionally adjusted for smoking, drinking, exercise, tooth brushing, and dental flossing

Bold indicates statistical significance (*p* < 0.05)

When HDL, as a main outcome, was used for calculation with the group means of bacterial burden, the power of this study exceeded 0.80 as the sample size ranged from 212 to 556 in two-sided tests (not shown).

## Discussion

Periodontal health and disease status have not been viewed in the context of a single periodontal pathogen, such as *P. gingivalis*, but rather in terms of the total pathogenicity of a biofilm [25]. The accumulated total burden of the harbored bacterial species has been suggested to complicate the bacterial community and is involved in the microbial shift from symbiosis to dysbiosis [26]. In the present study, persons who harbored all of the 4 different species showed poor HDL values after adjustment for clinically defined periodontitis and other potential behavioral factors. Additionally, the combination of harboring periodontal bacteria and periodontitis on decreased HDL and increased TG showed significant trends. Our results provide important epidemiological evidence that the accumulated burden of periodontal bacteria from saliva can independently affect regulation of HDL and TG levels, which are critical risk factors for atherosclerotic diseases, regardless of periodontitis. Such findings have not been reported previously, even though it has been shown that the presence of periodontitis, as defined by typical clinical measures, can contribute to a decrease in serum HDL and increase in TG [14, 27, 28], as well as to the development of atherosclerosis-related cardiovascular disease [29].

Interest in the role of the total periodontal bacterial burden, rather than that of a specific pathogen, in development of cardiovascular disease has emerged from studies reporting only one or two periodontal bacteria in relation to the occurrence of atherogenesis [30] and coronary heart disease [31]. Several recent case−control studies [32–35] and a cohort study [1] have investigated the role of co-existing major periodontal bacteria in a cardiovascular disease event, and their findings have suggested that a higher inflammatory burden of periodontal bacteria could elevate the risk of stroke, myocardial infarction, and coronary artery disease.

In addition to the importance of the impact of bacterial exposure on the development of cardiovascular diseases as an end-point health outcome, it is vital to highlight biomarkers associated with atherosclerosis medication, such as serum lipid profiles, to gain a better understanding on how infection and/or inflammation induced by periodontal bacteria play a role in altering lipid metabolism. Previous reports have noted that patients with periodontitis diagnosed based on clinical parameters have a risk of dyslipidemia, such as decreased HDL, increased TG, and increased LDL [27, 29, 36]. In addition, control of periodontal inflammation by standard treatment was shown to improve HDL and LDL levels [37], and findings of a recent meta-analysis [38] indicated that periodontal treatment enhanced the levels of atherosclerotic biomarkers, including HDL, TG, and TC.

The present findings provide evidence suggesting that the total inflammation burden induced by exposure to major periodontal bacteria could induce dyslipidemia by lowering HDL and possibly increasing TG, after adjustment for periodontitis as a clinical parameter. The mean differences in HDL lowered by bacterial burden seem to be clinically small; thus, the effect of the oral pathogenic burden on low HDL or hyper-triglycerides beyond the normal range is unclear. However, we could not ignore this independent, albeit small effect from exposure to oral infection or inflammation, which may trigger the development of dyslipidemia and even atherosclerosis in individuals harboring several risk factors [14, 29]. A recent cross-sectional study [13] reported that the presence of *A. actinomycetemcomitans* and *P. gingivalis* reduced the plasma level of HDL, while another study [39], which used a scoring method for 3 pathogen antibodies, combined with herpes simplex virus, showed association with decreased HDL. In a Japanese prospective study [40], increased tooth brushing frequency caused improvement in hypertriglyceridemia. Consistent with the findings from previous studies, our findings established that inflammation related to oral bacteria contributes to deterioration of the lipid metabolic pathway, although some evidence regarding this association remains lacking. Nevertheless, an important strength of this study is empirical evidence showing a connection between the total burden of exposure to *P. intermedia, P. gingivalis, T. denticola,* and *T. forsythia*, detected from stimulated saliva, with decreased HDL and a trend for increasing TG in serum. Saliva seems to be the sample most readily obtainable from a number of subjects, and the bacterial burden, which can deflect the metabolic pathway, must be sufficient for bacterial detection in saliva (even though plaque specimens are a better medium for sensitive bacterial detection) [18, 24].

In this study, we exploited GLM models, with calculation of LSMs, although the level of lipids was not normally distributed, as we were concerned about the major limitation of transformed data (lack of a real value, which makes it difficult to interpret). We assume that the 4 lipid values were normally distributed, because the distribution of the lipids in the general population is normal and, furthermore, the implication of such a robust model is easier to understand.

This study had some limitations. Since it was cross-sectional in nature, we were not able to examine the causal relationship between exposure and outcome, and the interplay between periodontal inflammation and lipid dysregulation via host response reaction is complex. To improve our understanding of the role of the oral bacterial burden in lipid metabolism, well-designed studies, such as prospective or randomized controlled trials, are needed to verify these results. On the other hand, the sample size of this study was small, as the sample size calculation was implemented after the study had been finished, rather than prior to commencement, as this was an exploratory study. Based on the results of this study, statistical power was confirmed using final sample size whether the power was at least 0.80 or not. In addition, this study focused on only 4 primary bacterial species related to periodontitis, originating from gingival pockets. Nevertheless, other bacterial species have been found to be associated with the risk of systemic disease [12]; for this reason, pathogenic burden levels were classified as low, moderate, or high, instead of 0, 1–3, and 4. Despite this disadvantage, the present results suggest that a clinical bacterial test for the 4 well-known periodontal bacteria could be useful for screening patients with a greater risk of serum lipid profile deterioration and development of atherosclerotic disease. It is possible that oral microorganisms interact with each other; thus, each organism should be quantified to assess the individual contribution to the total pathogenic burden and modification of lipid metabolism. Moreover, no information was obtained about changed host responses, such as inflammatory serum cytokines, induced by periodontal infection and that resulted in lipid metabolic modification. Other variables may also not have been assessed, even though some crucial potential confounders were adjusted for in our statistical models. For instance, reduced chewing ability related to low number of remain teeth affects proper mastication, which also influences lipids metabolism negatively considering inclusion criteria (10 teeth and more in mouth). Finally, generalization of the study results may be limited, as only a small number of elderly Japanese persons living a metropolitan area were examined in this study. Further studies that consider host metabolic dysfunction after exposure to major periodontal pathogens, and that also include various populations, as well as the general population of Japan, with larger sample sizes, are necessary.

## Conclusions

The results of this research suggest that a higher burden of bacteria was independently associated with lower HDL in serum after adjustment for periodontitis and other potential risk factors. This could show a possible link between exposure to major periodontal bacteria, in terms of the total burden, and increased risk of dysregulation of serum lipid metabolism. Furthermore, screening for the presence of periodontal bacteria could be beneficial to detect lipid changes earlier than atherogenesis, thus reducing the risk for development of atherosclerotic disease. Future studies that provide additional evidence about these issues are needed.

## Abbreviations

*A. actionomycetemconitans*: *Aggregatibacter actinomycetemcomitans*
ANOVA: One-way Analysis of Variance
BMI: Body mass index
CPI: Community Periodontal Index
DBP: Diastolic blood pressure
DMFT: Decayed, Missing, and Filled Teeth
GLM: General linear models
HDL: High-density lipoprotein
LDL: Low-density lipoprotein
LSM: Least square mean
*P. gingivalis*: *Porphyromonas gingivalis*
*P. intermedia*: *Prevotella intermedia*
PCR: Polymerase Chain Reaction
SBP: Systolic blood pressure
*T. denticola*: *Treponema denticola*
*T. forsythia*: *Tannerella forsythia*
TC: Total cholesterol
TG: Triglycerides
